# Ultrafast Cancer Cells Imaging for Liquid Biopsy via Dynamic Self-Assembling Fluorescent Nanoclusters

**DOI:** 10.3390/bios13060602

**Published:** 2023-05-31

**Authors:** Jinpeng Wang, Qingxiu Xia, Ke Huang, Lihong Yin, Hui Jiang, Xiaohui Liu, Xuemei Wang

**Affiliations:** 1 State Key Laboratory of Digital Medical Engineering, School of Biological Science and Medical Engineering, Southeast University, Nanjing 210096, China; 220202123@seu.edu.cn (J.W.); 220206125@seu.edu.cn (K.H.); sungi@seu.edu.cn (H.J.); 2 Key Laboratory of Environmental Medicine Engineering, Ministry of Education, School of Public Health, Southeast University, Nanjing 210009, China; 220213995@seu.edu.cn (Q.X.); lhyin@seu.edu.cn (L.Y.)

**Keywords:** in situ self-assembly, ultrafast cancer cells imaging, fluorescent nanoclusters, lung cancer, liquid biopsy

## Abstract

Lung cancer-specific clinical specimens, such as alveolar lavage fluid, are typically identified by microscopic biopsy, which has limited specificity and sensitivity and is highly susceptible to human manipulation. In this work, we present an ultrafast, specific, and accurate cancer cell imaging strategy based on dynamically self-assembling fluorescent nanoclusters. The presented imaging strategy can be used as an alternative or a complement to microscopic biopsy. First, we applied this strategy to detect lung cancer cells, and established an imaging method that can rapidly, specifically, and accurately distinguish lung cancer cells (e.g., A549, HepG2, MCF-7, Hela) from normal cells (e.g., Beas-2B, L02) in 1 min. In addition, we demonstrated that the dynamic self-assembly process that fluorescent nanoclusters formed by HAuCl_4_ and DNA are first generated at the cell membrane and then gradually enter the cytoplasm of lung cancer cells in 10 min. In addition, we validated that our method enables the rapid and accurate imaging of cancer cells in alveolar lavage fluid samples from lung cancer patients, whereas no signal was observed in the normal human samples. These results indicate that the dynamic self-assembling fluorescent nanoclusters-based cancer cells imaging strategy could be an effective non-invasive technique for ultrafast and accurate cancer bioimaging during liquid biopsy, thus providing a safe and promising cancer diagnostic platform for cancer therapy.

## 1. Introduction

With the popularity of CT, a large number of patients with lung nodules have been detected, but these nodules are difficult to give an accurate judgment by imaging, and only about 3.8% of these examined lung nodules are malignant nodules, so the accurate judgment of benign and malignant has been an international problem in the early diagnosis of lung cancer [1]. To compensate for the limitations of CT, bronchoscopic lung biopsy can not only detect lesions in the bronchial lumen that cannot be detected by chest CT [2], but also sample for pathogenesis and perform biopsy to clarify the nature of the lesions and obtain a more accurate diagnosis [3]. Bronchoscopic lung biopsy includes liquid biopsy (bronchoalveolar lavage fluid, BALF), cytobrush, and tissue biopsy [4].

Bronchoalveolar lavage fluid is obtained during bronchoscopy [5]. Because of its proximity to tumor tissue, it has a higher sensitivity for suspected lung disease, especially locally advanced non-metastatic lung cancer, than other fluids such as plasma or pleural fluid. Its collection requires a minimally invasive procedure that can be repeated with minimum risk [6]. Therefore, the use of local lavage fluid in the tumor area has significant advantages over the use of free DNA or circulating tumor cells in circulating blood for the diagnosis of tumor disease [7]. Malignant cells may be shed from adjacent lung tumors into the corresponding bronchoalveolar space and identified by cytology [8]. The concertional BALF cytology alone has a modest sensitivity for diagnosing lung cancer (between 29% and 69%), and is highly influenced by human operation [9]. Therefore, there is an urgent need for a rapid, specific, and accurate diagnostic method to compensate for the existing conventional cytological tests (BALF cytology).

With the study of tumor pathogenesis, gene methylation has increasingly gained prominence as a promising diagnostic technique for diagnosing molecular markers of cancer in bronchoalveolar lavage fluid [10]. Among a series of methylation abnormal genes associated with lung cancer, short stature homeobox 2 (SHOX2) is the most intensively studied and is an excellent indicator for the early diagnosis of lung cancer [11]. RFSSF1A is a Ras-related region family 1A antagonistic to Ras proto-oncogene, a typical tumor suppressor gene associated with gene transcription, signal transduction, cytoskeleton, cell cycle, cell adhesion, and apoptosis, and is closely related to lung adenocarcinoma development [12]. The molecular methylation test based on bronchoalveolar lavage fluid is more objective and sensitive compared with cytology, and can improve the stability of the test and significantly outperform traditional cytology, especially for patients with stage I and II lung cancer or early-stage micro nodules with tumors <1 cm, where the diagnostic sensitivity can reach more than 70% [13]. However, most of the current methylation detection methods are based on the fluorescent PCR method, which still cannot achieve rapid and immediate liquid biopsy for lung cancer patients.

Gold nanoclusters have many excellent properties, including unique catalytic activity, fluorescence properties, etc. [14]. In recent years, biological self-assembly strategies for the synthesis of fluorescent nanoprobes have been widely reported. The biosynthesis of fluorescent metal nanomaterials does not require the addition of chemical reagents as encapsulants to prevent the clustering of nanoprobes, and also enables the simultaneous real-time detection of target cells. In situ biological self-assembly is a specific process based on the internal tumor microenvironment, and it is mainly due to the difference with the normal cellular microenvironment [15]. It was shown that in tumors, gold, silver, and platinum plasma can be converted into high-performance fluorescent probes for the bioimaging of cancer cells [16,17]. The potential of these biosynthetic gold nanoclusters for in vivo and in vitro multimodal tumor bioimaging was successfully demonstrated by introducing chloroauric acid (HAuCl_4_) into cancer cells and transplanting the tumor mouse model [18,19,20]. In recent years, our group has used metal ions to mediate the in-situ synthesis of highly sensitive and specific fluorescent probes for cancer cells imaging [21,22,23]. Recently, localized surface plasmon resonance biosensors with self-assembling gold nanoislands (SAM-AuNIs) and polyphenol-metal three-dimensional network-based methods have been reported to detect and distinguish exosomes in plasma samples, showing that the self-assembling nanomaterials will be a label-free, universal, low-cost, and easy-to-scale-up technique for liquid biopsy in cancer diagnosis [24,25]. In addition, fluorescent probes have proven to be indispensable chemical tools in the fields of chemical biology and medicine. The ability to detect intracellular species and to monitor physiological processes has not only advanced our knowledge of biology, but has also opened up new approaches to the diagnosis of disease [26,27].

Here, we report an ultrafast dynamic self-assembling fluorescent nanoclusters-based cancer cells imaging strategy for the identification of lung cancer cells. This strategy can quickly and accurately discriminate between cancer cells (e.g., A549, HepG2, MCF-7, Hela) and normal cells (e.g., Beas-2B, L02) in 1 min. In addition, we were able to observe and verify the dynamic process from the tumor cell membrane to the cytoplasm under real-time fluorescence microscopy. Finally, we validated that this strategy enables a rapid and accurate imaging of cancer cells in alveolar lavage fluid from lung cancer patients. More importantly, this ultrafast, specific strategy is expected to be applied to the accurate diagnosis of other tumor cells during liquid biopsy.

## 2. Materials and Methods

### 2.1. Cell Culture

Beas-2B and A549 cells (~2 × 10^5^ cells/mL concentration) were cultured in Dulbecco’s modified Eagle’s medium (Beijing Solarbio Science & Technology Co., Ltd., Beijing, China) supplemented with 10% fetal bovine serum (Beijing Solarbio Science & Technology Co., Ltd., Beijing, China). The cells were incubated at 37 °C in 5% CO_2_ until they reached 90% confluence. After harvesting the cells with 0.05% Trypsin (Thermo Fisher Scientific Inc., Waltham, MA, USA), the cells were centrifuged at 1000 rpm for 3 min. The supernatant was then removed into confocal dishes (20 mm) (Wuxi NEST Biotechnology Co., LTD., Wuxi, China), and fresh cell medium (1 mL) was added to resuspend the cell at a cell concentration of 1 × 10^5^ cells/mL.

### 2.2. Characterization of Self-Assembling Fluorescent Nanoclusters

Previous studies have shown that DNA (Herring essence DNA, <500 bp; Solarbio) can enhance in situ self-assembly fluorescence. This is mainly due to the double-stranded structure of the DNA binding to the metal ions during the reaction. This allows access to the cell through the phospholipid bilayer on the cell surface. Pre-experiments were performed to select the best concentration of HAuCl_4_. From the results, the concentration of 250 μM is more satisfactory. This ensures stronger fluorescence and less damage to the cells. When A549 cells adhered, HAuCl_4_ solution was added, and it was ensured that the final concentration was 250 μM. Next, the DNA was added to the medium, and it was ensured that the concentration was 2 μg/mL. After incubation of A549 cells with HAuCl_4_ solution and DNA for 2 h, the medium was discarded. Beas-2B cells were treated as above. Then, we used the real-time acquisition (time series function) of the Zeiss microscope, set the time interval for acquisition to 10 s, and observed the fluorescence dynamic changes.

### 2.3. In Situ Injection of Fluorescent Nanoclusters Precursors

A fully automated microinjector (Eppendorf FemtoJet 4i/FemtoJet 4x; InjectMan^®^ 4; Hamburg, Germany) was used to inject HAuCl_4_ and DNA into cells. Sample preparation and handling are crucial for successful microinjection and must be performed with great care. To prevent blocking of the injection capillaries, HAuCl_4_ and DNA solution should be filtered through 0.22 µm pore-size filters. Microinjection capillaries are typically made from 1 mm outer diameter borosilicate glass. One end of the capillary is drawn to a fine tip with an opening size or inner diameter of 1 µm [28,29]. A549 was selected and the above-treated cells were taken for the experiment at a cell density of approximately 60–70%. The concentration of the injection solution was the same as that of self-assembling. The experimental parameters were set to pi (injection pressure) = 120 hPa; ti (injection time) = 0.4 s; pc (compensation pressure) = 30 hPa. The mixed solution was then injected into the A549 using this parameter. The fluorescence reaction of the cells was observed under a Zeiss (Jena, Germany) microscope.

### 2.4. Subcellular Structure Metal Content Analysis

To determine the distribution of metal elements within the cellular subcellular structures, metal ions were determined by ICP mass spectrometry. A549 cells were collected after the above treatment, and the number of cells was 1 × 10^6^. HAuCl_4_ (250 μM) and DNA (2 μg/mL) were added to the cells and incubated for 2 h. The cells were then subjected to differential centrifugation [30]. Centrifugation steps were performed as described in the literature. Depending on the size of the subcellular structures, they were centrifuged into different samples [31].

### 2.5. Diagnostic Imaging of Clinical Samples

Clinical samples of alveolar lavage fluid were obtained from 2 lung cancer patients and 2 healthy donors at Zhongda Hospital Southeast University. Alveolar lavage samples were stored at 4 °C after collection and processed by the laboratory immediately upon receipt. A volume of 100 µL of HAuCl4 (2.50 mM) and 100 µL of herring essence DNA (20 µg/mL) were added to each clinical sample (800 µL), and the final concentrations of HAuCl4 and herring essence DNA were 250 µM and 2 µg/mL, respectively. Afterwards, they were placed under the Zeiss microscope. Then, the real-time imaging function of the Zeiss microscope was used for observation. The experimental results were recorded and the experimental data were processed.

## 3. Results and Discussion

### 3.1. Characterization of Dynamic Self-Assembling Fluorescent Nanoclusters for Ultrafast Cancer Cells Imaging

**Scheme 1:**
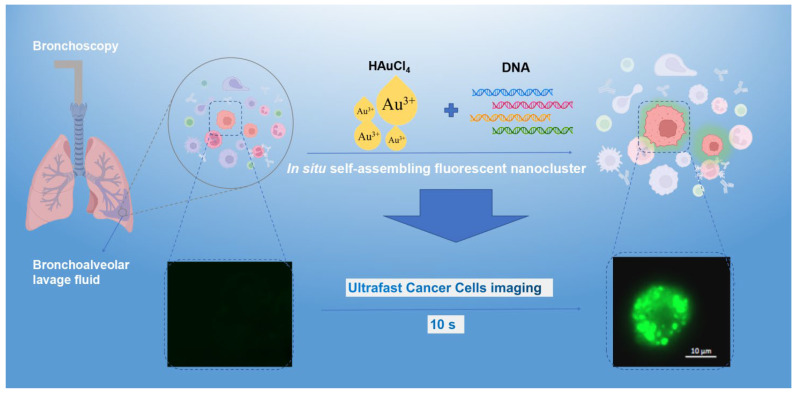
Schematic diagram of ultrafast cancer cell imaging based on dynamic self-assembled fluorescent nanoclusters strategy.

**Figure 1:**
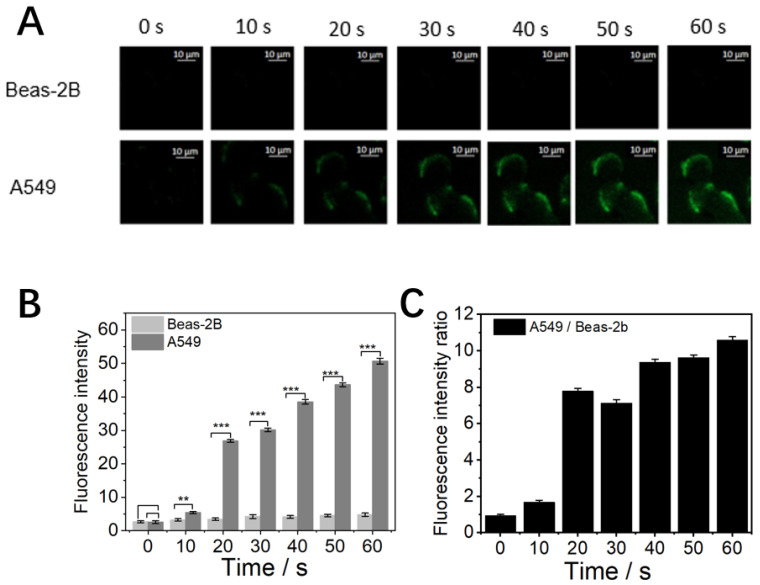
Fluorescence changes in normal and tumor cells after addition of gold for in situ synthesis (250 µM). (**A**) Plot of cell fluorescence changes (in one minute). (**B**) Histogram of cellular fluorescence intensity. (**C**) Histogram of the ratio of fluorescence intensity of normal cells to tumor cells.

The principle of ultrafast cancer cell imaging based on a dynamic self-assembled fluorescent nanocluster strategy is shown in Scheme 1. Previous studies have shown that DNA can facilitate in situ synthesis. From the result of this work (Figure S1), it is clear that the fluorescence intensity of the combination group with the addition of HAuCl_4_ and DNA is stronger than HAuCl_4_ alone. From the results of the pre-experiment (Figure S2), we determined the subsequent HAuCl_4_ concentration to be 250 μM. In this work, we confirmed the time course of the formation of self-assembling fluorescent nanoclusters and the subsequent spatial changes using fluorescence microscopy under a dynamic filming model. After adding the same concentration of metal ions, we observed the change of fluorescence dynamics (Figure 1A). As shown in Figure 1A, both A549 and A549/DDP displayed the same fluorescence intensity with time change after 10s. After that, the fluorescence intensity increased slowly until it stabilized after about 30 min (Figure S3). From the histogram (Figure 1B), there was a significant difference between the experimental and control groups after 10 s (The asterisk indicates significance *p* < 0.01). After 20 s, the difference gradually increased and became more significant (The asterisk indicates significance *p* < 0.001). At 60 s, the fluorescence intensity of the experimental group increased with time, and it was found that the fluorescence was mainly produced on the cell membrane. We speculate that metal ions are likely to generate fluorescent nanoclusters through redox reactions with chelated membrane surface proteins and reactive oxygen species. The blank control (Beas-2B cells) did not change in fluorescence intensity over time. As can be seen from the graph (Figure 1C), the fluorescence ratio of tumor cells to normal cells increases significantly at 20 s, demonstrating that the dynamic self-assembly fluorescent nanoclusters strategy is ultrafast and highly specific.

### 3.2. Characterization of the Spatial Distribution of Fluorescent Nanoclusters

**Figure 2:**
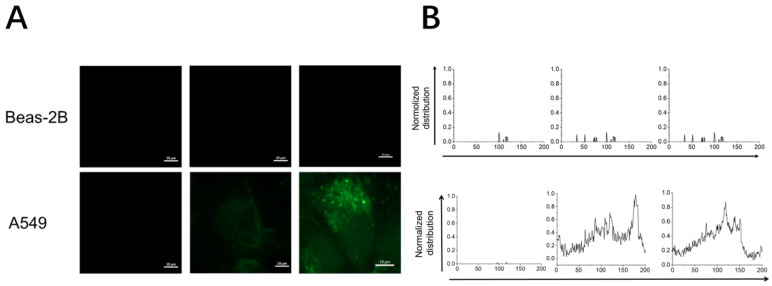
Spatial characterization of the fluorescence distribution of single cells generated by in situ synthesis. (**A**) Single cell fluorescence change graph. (**B**) Discounted spatial distribution of fluorescence intensity.

It is clear that in situ synthesis can achieve fluorescence imaging in seconds based on the above experiments. According to the fluorescence distribution diagram (Figure 2A), at 1 min, the A549 cell fluorescence mainly appeared on the cell membrane. The intensity of cellular fluorescence gradually increased with time, and at 10 min, the cellular fluorescence was mainly concentrated in the cytoplasm. By calculating the fluorescence at each point of the cell lateral, Beas-2b cells are essentially non-fluorescent. Fluorescence fluctuations were observed in A549 cells, with two intensity peaks at 1 min, indicating that fluorescence was mainly concentrated in the cell membrane at this time. At 10 min, the fluorescence fluctuation has a peak in the middle of the cell, which indicates that the fluorescence is mainly concentrated inside the cell at this time (Figure 2B). We followed up with additional experiments and found that for unadhered tumor cells, the fluorescence trend was similar (Figure S4).

**Figure 3:**
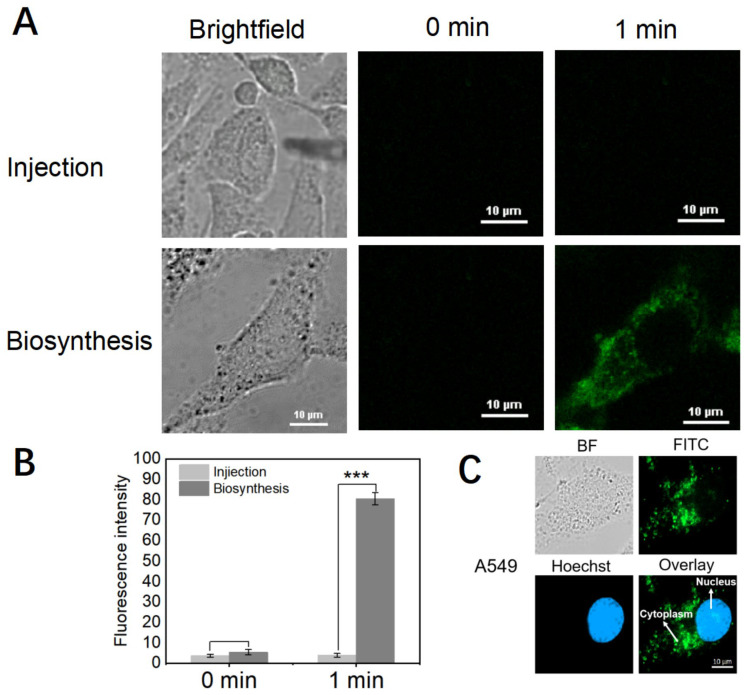
(**A**) Fluorescence characterization of microinjection and in situ synthesis. (**B**) Fluorescence intensity comparison histogram. (**C**) Fluorescence results from Honchest co-staining experiments.

As time changed, the fluorescent area no longer changed. To confirm the site of cellular fluorescence production, A549 cells were taken, followed by co-incubation with Hoechst (1:1000) and HAuCl_4_ (250 µM) solution for 15 min. As can be seen from the figure (Figure 3C), the blue fluorescence produced by Hoechst is mainly concentrated in the nucleus, and green fluorescence from in situ self-assembly is mainly concentrated in the cytoplasm. The basic process of in situ self-assembly reaction can be deduced, where metal ions pass through the cell membrane and enter the cytoplasm, where they continue to react to form more fluorescent nanoclusters. As gold nanoclusters continue to be produced, they can cause some damage to the cell and, thus, alter the permeability of the cell membrane. This also allows more gold ions to enter the membrane, resulting in a stronger in situ synthesis, leading to a gradual increase in fluorescence and, ultimately, cell death.

The importance of the cell membrane in situ self-assembly was then demonstrated by using a fully automated microinjection device. To verify such a conclusion, two groups of A549 cells in the same condition were selected(group 1 and group 2), and group 1 was injected with HAuCl_4_ (250 μM) and DNA (2 µg/mL) solution directly into the cells without passing through the cell membrane. HAuCl_4_ (250 μM) and DNA (2 µg/mL) solutions were added to group 2. In this experiment, micron-injection devices are used to avoid large damage to the cells. As can be seen from the figure (Figure 3A), the cell fluorescence did not change after injection using the micro-injection device, in contrast with in situ self-assembled fluorescence. Additionally, the fluorescence intensity was significantly different compared to the in situ synthesis, with a significant difference in cell fluorescence at 1 min (Figure 3B, The asterisk indicates significance *p* < 0.001). This also reveals the role of the cell membrane in in situ synthesis and it only occurs when metal ions enter the cell through the cell membrane.

### 3.3. Analysis of Clinical Samples

**Figure 4:**
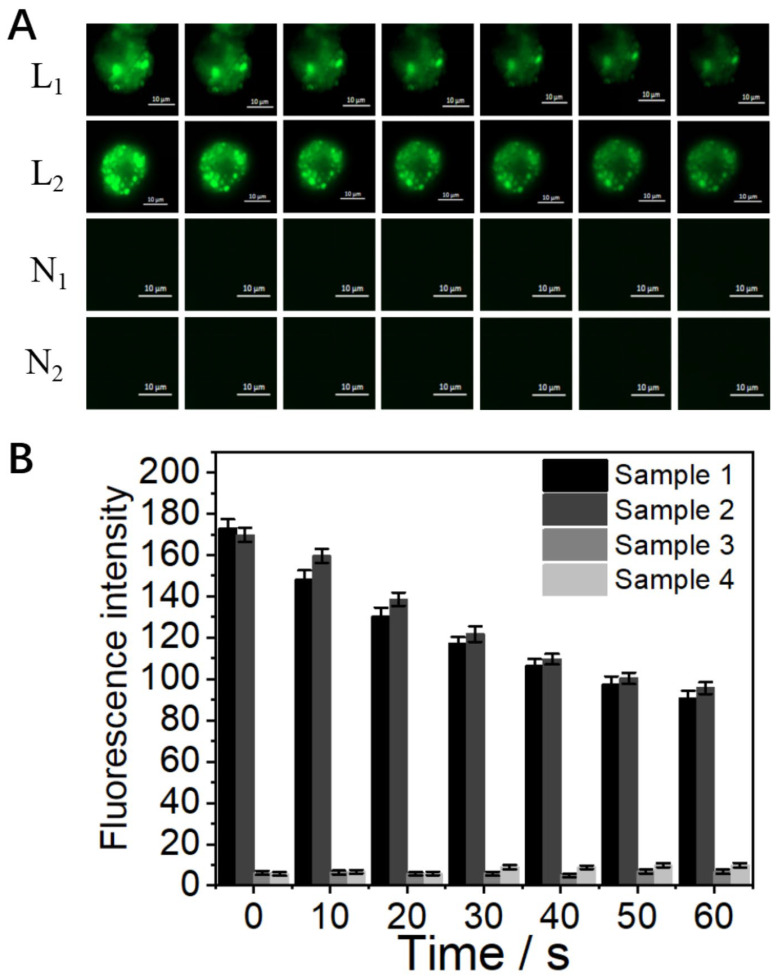
Fluorescence images of in situ synthesis results for clinical samples from two lung cancer patients (L) and two healthy donors (N). (**A**) Fluorescence kinetics of four clinical samples within one minute after the addition of HAuCl_4_ (250 μM) and DNA (2 μg/mL). (**B**) Histogram of fluorescence intensity of clinical samples.

Clinical samples of alveolar lavage fluid have a more complex microenvironment than cellular samples, which makes them more challenging to assay. No fluorescence was produced in the two healthy donors. In the two lung cancer patients, fluorescence appeared at 10 s, followed by a gradual decrease in fluorescence intensity over 1 min (Figure 4A). As can be seen from the figure (Figure 4B), there was a large difference in fluorescence intensity between the control and experimental groups. Then, we observed the change in fluorescence intensity of the experimental group from 1 to 60 min; the trend of fluorescence change was weakening at first and then gradually stabilizing. However, it is worth noting that the fluorescence intensity of the clinical samples was strongest at the beginning, then slowly weakened over time and eventually stabilized. This is different from previous cellular experiments. We believe that due to the complexity of the clinical sample, the addition of the gold (HAuCl_4_) precursor results in a rapid in situ self-assembly reaction, producing a large number of fluorescent metal nanoclusters, whereas the cells in the sample are killed over time, resulting in a slow weakening of the fluorescence intensity. Nevertheless, the experimental results of clinical samples also demonstrate the ultra-high speed of in situ self-assembly itself, which allows for the detection of tumor cells in seconds.

## 4. Conclusions

The cancer cell imaging strategy presented in this study has the ability to quickly and accurately discriminate between cancer cells (e.g., A549, A549/DDP) and normal cells (e.g., Beas-2B) in 1 min. In addition, the dynamic process from the tumor cell membrane to the cytoplasm was observed and verified under real-time fluorescence microscopy. Meanwhile, we validated that this strategy enables the rapid and accurate imaging of cancer cells in alveolar lavage fluid from lung cancer patients. More importantly, our study demonstrates that the cell membrane plays a critical role in the process of in situ self-assembly. In conclusion, the dynamic self-assembly fluorescent nanoclusters-based cancer cell imaging strategy would be an effective noninvasive technique for ultrafast and accurate cancer bioimaging during liquid biopsy, thus providing a safe and promising cancer diagnostic platform for cancer therapy.

## Data Availability

The data presented in this study are available on request from the corresponding author.

## Supplementary Materials

The following supporting information can be downloaded at: https://www.mdpi.com/article/10.3390/bios13060602/s1. Figure S1: Dynamic self-assembly of fluorescent nanoclusters in Beas-2B and A549 cell lines; Figure S2: Optimization of experimental conditions based on different concentrations of chloroauric acid in A549 cell lines; Figure S3: Self-assembly of fluorescent nanoclusters fluorescent nanoclusters from 1 min to 60 min in in Beas-2B and A549 cell lines; Figure S4: Fluorescence images of dynamically self-assembled fluorescent nanoclusters in A549 cells in the state of suspension; Figure S5: Characterization of dynamic self-assembled nanofluorescent cluster distribution on subcellular organelles in A459 cell line; Figure S6: Self-assembly of fluorescent nanoclusters in L02, HepG2, MCF-7, Hela cell lines.
