# Supplementary material for: Ultrafast Cancer Cells Imaging for Liquid Biopsy via Dynamic Self-Assembling Fluorescent Nanoclusters
Source: Biosensors (Basel). 2023 May 31;13(6):602. doi: 10.3390/bios13060602 (PMC10296153; doi:10.3390/bios13060602)
Supplement: Supplementary file 1 [file biosensors-13-00602-s001.zip › biosensors-2367832-supplementary.pdf]

Supporting information

Ultrafast Cancer Cells Imaging for Liquid Biopsy via Dynamic Self-Assembling Fluorescent Nanocluster

Jinpeng Wang[1, #], Qingxiu Xia[2, #], Ke Huang,[1] Lihong Yin[2], Hui Jiang[1], Xiaohui Liu[1*], Xuemei Wang[1*]

[1]*State Key Laboratory of Digital Medical Engineering, School of Biological Science and Medical Engineering, Southeast University, Nanjing 210096, China.*

[2]*Key Laboratory of Environmental Medicine Engineering of Ministry of Education, School of Public Health, Southeast University, Nanjing 210009, China.*

[#] Contributed equally.

*Correspondence: 101013182@seu.edu.cn (X.H. Liu); xuewang@seu.edu.cn (X.M. Wang)

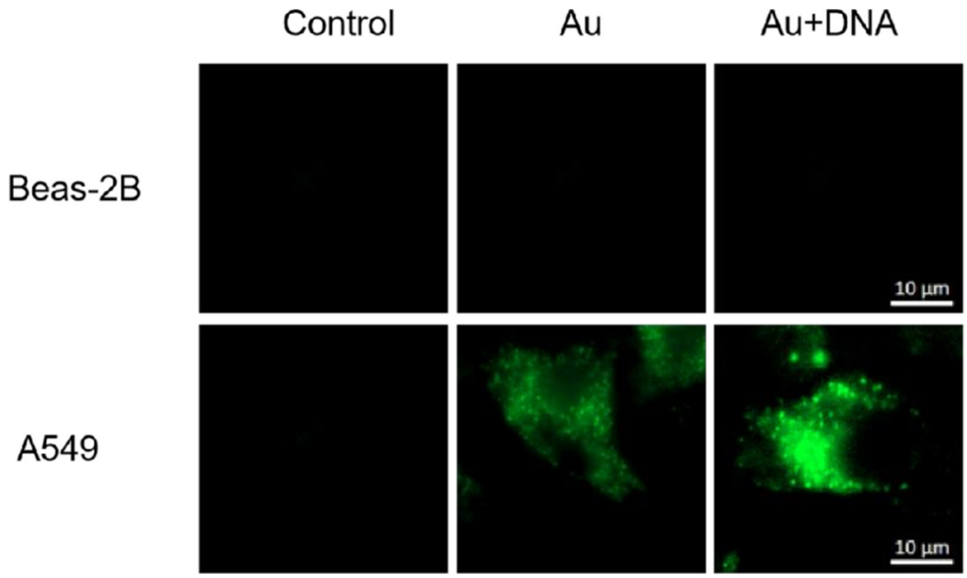

Figure S1.**Dynamic self-assembly of fluorescent nanoclusters in Beas-2B and A549 cell lines.**

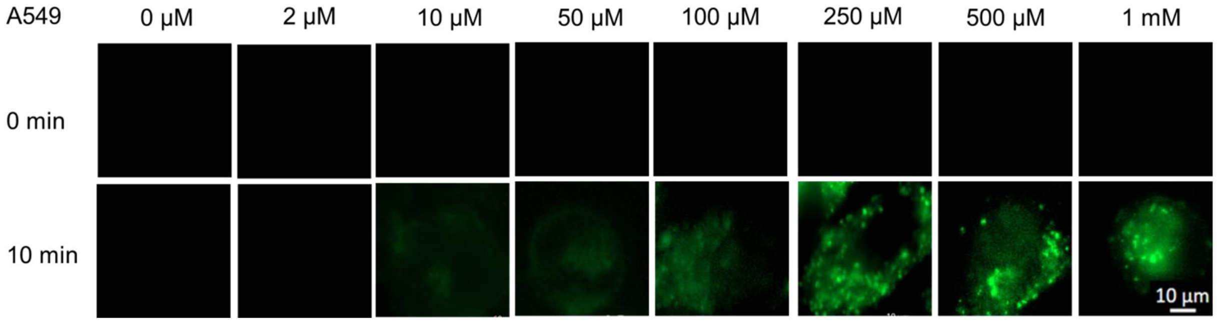

Figure S2. **Optimization of experimental conditions based on different concentrations of chloroauric acid in A549 23 cell lines.**

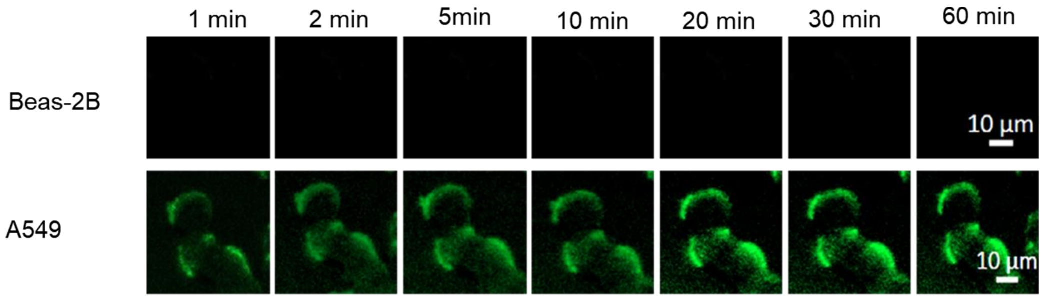

Figure S3. **Self-assembly of fluorescent nanoclusters fluorescent nanoclusters from 1 min to 60 min in in Beas-2B 26 and A549 cell lines.**

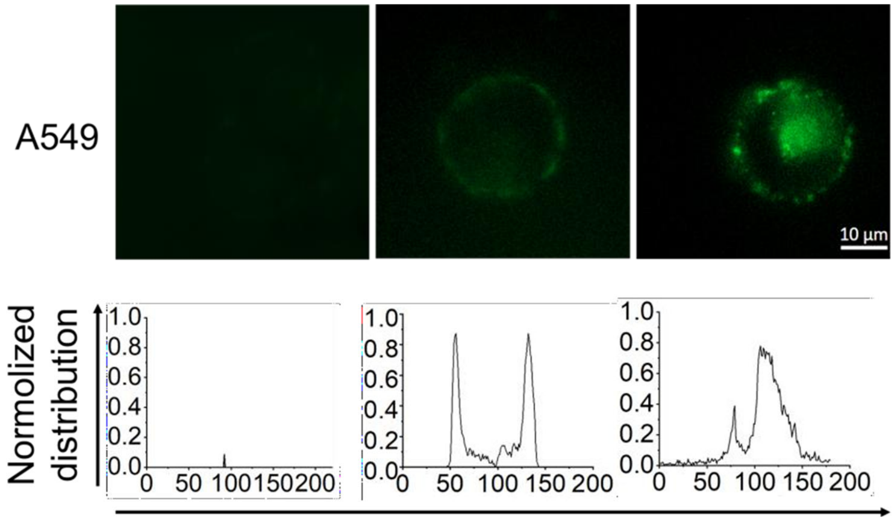

Figure S4. **Fluorescence images of dynamically self-assembled fluorescent nanoclusters in A549 cells in the state of suspension.**

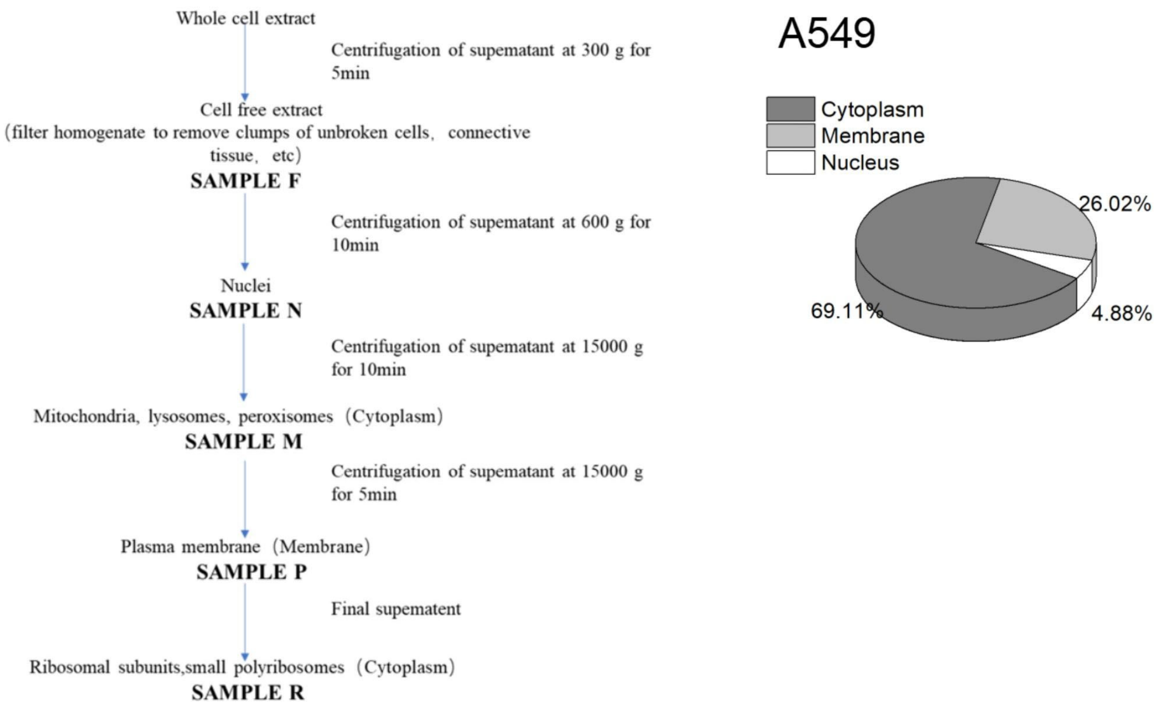

Figure S5. **Characterization of dynamic self-assembled nanofluorescent cluster distribution on subcellular 35 organelles in A459 cell line.**

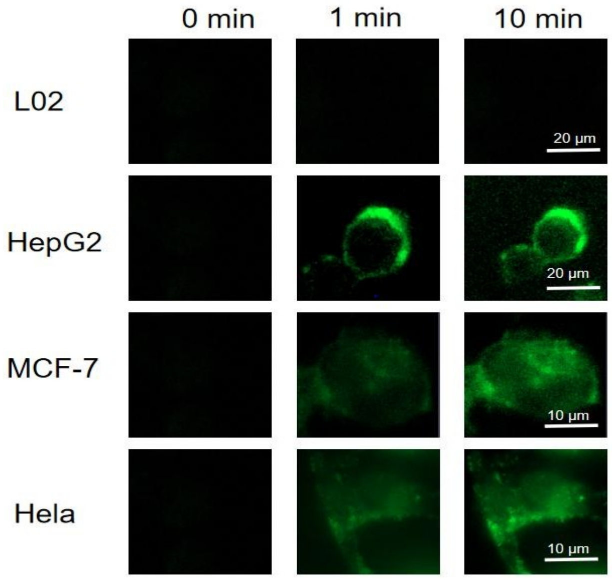

Figure S6. Self-assembly of fluorescent nanoclusters in L02, HepG2, MCF-7, Hela cell lines.
